# Validation of NINDS-CSN neuropsychological battery for vascular cognitive impairment in Chinese stroke patients

**DOI:** 10.1186/s12883-015-0270-z

**Published:** 2015-03-03

**Authors:** Xiangliang Chen, Adrian Wong, Ruidong Ye, Lulu Xiao, Zhaojun Wang, Ying Lin, Fang Yang, Hua Li, Ting Feng, Lihui Duan, Yunfei Han, Qiliang Dai, Juan Du, Gelin Xu, Vincent Mok, Yunyun Xiong, Xinfeng Liu

**Affiliations:** Department of Neurology, Jinling Hospital, Medical School of Nanjing University, Nanjing, 210002, Jiangsu China; Department of Medicine and Therapeutics, The Chinese University of Hong Kong, Shatin, Hong Kong SAR, China

**Keywords:** Vascular cognitive impairment, Stroke, Neuropsychology, Validation study, China

## Abstract

**Background:**

The NINDS-Canadian Stroke Network (NINDS-CSN) recommended a neuropsychological battery of three protocols to diagnose vascular cognitive impairment (VCI), however, due to culture and language differences, the battery cannot be directly used in China. Validation of the battery in mandarin Chinese is lacking. Our study investigated the reliability and validity of the adapted Chinese versions of the battery in stroke patients with high probability of VCI.

**Methods:**

Fifty mild stroke patients (median of National Institute of Health Stroke Scale [NIHSS] score, 2) and 50 stroke-free normal controls were recruited. All subjects’ demographics, clinical history, and stroke severity were recorded. The NINDS-CSN neuropsychological protocols were adapted into the Chinese versions. External validity, defined as the ability of the protocol summary scores to differentiate stroke patients from controls, was determined using the area under the curve (AUC) of the receiver operating characteristics curve. We also evaluated internal consistency and intra-rater reliability.

**Results:**

Stroke patients performed significantly poorer than controls on all three protocols (F statistics between 24.9 and 31.4, *P* < 0.001). External validity evaluated by AUCs was 0.88 (95% confidence interval [CI], 0.81-0.95), 0.88 (95% CI, 0.81-0.94), and 0.86 (95% CI, 0.79-0.94) for the 60-min, 30-min and 5-min protocols, respectively. Cronbach’s alpha of the cognitive tests was 0.87 for all subjects. Intra-rater reliability was acceptable with intraclass correlation coefficients 0.90, 0.83 and 0.75 for the 60-min, 30-min and 5-min protocols, respectively.

**Conclusions:**

The adapted Chinese versions of three NINDS-CSN neuropsychological protocols were valid and reliable for assessing VCI in Chinese patients with mild stroke.

## Background

Vascular cognitive impairment (VCI) represents the spectrum of cognitive impairment associated with evident stroke or subclinical vascular brain injury [1]. Around 2/3 of stroke patients may suffer from VCI [2,3], consequently they are more prone to have decline of quality of life [4], depression [5], and poor survival [6]. Considering the clinical importance of VCI, the National Institute for Neurological Disorders and Stroke and Canadian Stroke Network (NINDS-CSN) recommended a neuropsychological battery of three protocols (60-min, 30-min and 5-min) for early identification and diagnosis of VCI [7]. Because there are language and culture differences across countries, validation of the NINDS-CSN neuropsychological protocols is critical for the implementation of VCI diagnosis. Currently, Korean [3], Hong Kong [8], France [9] and Singapore [10] have validated these protocols in stroke or transient ischemic attack patients. However, there is no validation study in the population of mainland China, where stroke and dementia burden are one of the highest countries worldwide [11,12].

Considering that stroke patients had high probability of VCI after 3 months post-stroke, we developed a Chinese adaption for the NINDS-CSN battery, and aimed to investigate the external validity and reliability of the adapted Chinese versions for assessing VCI in post-stroke patients.

## Methods

### Subjects

Our study was a case–control study. Cases were post-stroke patients who fulfilled the following criteria: aged 50 years or older; patients with an evident ischemic stroke at least 3 months before; absent of traumatic brain injury, Parkinson disease, or psychiatric disorders known to influence cognitive function; and without motor, sensory or speech impediment hindering their participation in cognitive tests. Stroke patients were recruited at the out-patient clinic in a teaching hospital in Nanjing, China. An available informant who was knowledgeable of the patient’s cognitive status was also required. Patients with a history of hemorrhagic stroke, a pre-existing diagnosis of dementia, or a Mini-Mental State Examination (MMSE) score ≤10 were excluded [13].

Controls were recruited through advertisement on bulletin boards at our out-patient clinic and in a community; they were enrolled if they scored >24 on the MMSE at screening without historical records of stroke or transient ischemic attack. We attempted to match cases and controls on age and sex. All subjects gave written informed consents and the study was approved by the institutional review board at Jinling Hospital.

We collected all subjects’ demographic data (age, sex, education, and handedness), clinical history (stroke, transient ischemic attack, myocardial infarction, atrial fibrillation, hypertension, hyperlipidemia, diabetes mellitus, past smoking and alcohol abuse), and physical examinations (body mass index, blood pressure, and stroke severity measured by National Institutes of Health Stroke Scale [NIHSS]).

### Neuropsychological protocols

Three VCI neuropsychological protocols were recommended by the NINDS-CSN, with different protocols serving different purposes [7]. The 60-min protocol was developed for studies that required a breakdown of cognitive functions by domain; and the four tested domains were as follows: executive/activation, language, visuospatial, and memory. In addition, neuropsychiatric/depressive symptoms were assessed using the neuropsychiatric inventory questionnaire [14] and the geriatric depression scale [15]; Tests of the 30-min protocol were selected within the 60-min protocol for clinical screening, including executive/activation and memory domains. The 5-min protocol was designed as a quick-screening tool, consisting of selected subtests from the Montreal Cognitive Assessment (MoCA)-a 5-word immediate, delayed and recognition memory test, a 6-item orientation task and an animal naming test. Total score was calculated in the same way as Wong et al. study [8]. The tests and scales that composed the adapted Chinese versions of NINDS-CSN neuropsychological protocols were shown in Table 1.

**Table 1:** **Adapted Chinese versions of NINDS-CSN neuropsychological protocols**

**Cognitive tests**	**60-min**	**30-min**	**5-min**
**Executive/activation**			
ANT [16]	√	√	
WAIS-III Digit symbol-coding test [17]	√	√	MoCA subtests [7]
TMT A [18]	√	-	5’- Immediate recall
TMT B [18]	√	-	5’- Delayed recall
Language			5’- Recognition
Modified BNT [19]	√	-	6’- Orientation
Visuospatial			9’- ANT
RCFT copy [20]	√	-	
Memory			
HVLT-R delayed recall [21]	√	√	
RCFT delayed recall [20]	√	-	
Neuropsychiatric/depressive symptoms			
NPI-Q [14]	-	-	
GDS [15]	-	-	

*Abbreviations*: *ANT* Animal naming test, *TMT* Trail making test, *BNT* Boston naming test, *RCFT* Rey-Osterrieth Complex Figure Test, *HVLT-R* Revised Hopkins verbal learning test, *NPI-Q* Neuropsychiatric Inventory questionnaire, *GDS* Geriatric Depression Scale.

For ease of administration, we adapted the neuropsychological protocols into Chinese versions. For trail making test (TMT) B, where the individual is required to draw lines alternately between numbers and letters, the English letters were replaced by Chinese characters according to the “heavenly stems and earthly branches”. The Hopkins verbal learning test was adapted with reference to the Chinese frequency list. One of the items “Opal” was uncommon in Chinese, which was changed to “diamond”. Modifications were also made to the Boston naming test (BNT). Based on the 15-item version, two items with low frequency in Chinese (“octopus” and “beaver”) were replaced by Chinese alternatives “sea horse” and “mouse”.

Two supplemental tests-the Chinese version of MMSE and MoCA, Beijing version (MoCA-BJ) were also evaluated in our study. Subjects were considered cognitively impaired using cut-off points of MMSE as 17/18 for illiterates, 19/20 for individuals with 1 to 6 years of education, and 24/25 for those with 7 or more years of education [22,23]; and the corresponding cut-off points of MoCA were 13/14, 19/20 and 24/25 for illiterates, individuals with 1 to 6 years of education, and those with 7 or more years of education, respectively [24].

### Statistical analysis

To compare demographic and clinical data of stroke patients and controls, independent sample *t* test for normal distributed continuous data, χ^*2*^ tests or Fisher’s exact tests for categorical data, and trend test for ordinal data were used as appropriate. Effect sizes were calculated with Cohen’s *d* tests. Cohen’s *d* effect sizes of 0.2, 0.5, and 0.8 were considered small, medium, and large, respectively [25].

To determine the external validity of three adapted Chinese versions of NINDS-CSN protocols, tests scores were converted to standardized z scores. TMT time scores were multiplied by −1 after standardization. Averaged z scores were calculated by the 60-, 30-, and 5-min protocols. Z scores between stroke patients and controls on each protocol were compared with education adjusted. Receiver operating characteristic (ROC) curve analyses with area under the curve (AUC) were used to define how well the three protocols differentiated stroke patients from controls. An AUC of 50% corresponds to a random classification and 100% a perfect classification [26]. Meanwhile, to evaluate MoCA-BJ and MMSE as a screening tool in stroke patients, the Kappa statistic was used to assess test agreement [27] with the 60-min protocol. Patients were considered cognitively impaired if they performed 1.5 SDs below the control mean on at least one cognitive domain [1].

Reliability of the protocols was assessed by the internal consistency between individual cognitive tests. The intra-rater reliability was evaluated using intraclass correlation coefficients (ICC) [28]. The internal consistency for cognitive tests was estimated by the Cronbach’s alpha statistic [29]. A two-sided *P* value of less than 0.05 was considered to indicate statistical significance. All statistical analyses were performed with the use of SPSS Statistics for Windows, version 17.0.

## Results

Fifty stroke patients and 50 controls were recruited. In stroke patients, the median NIHSS score on admission was 2 (interquartile range [IQR], 0–4); the median interval between stroke onset and cognitive assessment was 206 days (IQR, 94 days-272 days). The median consuming time was 1.2 h (IQR, 0.9 h-1.5 h).

The clinical characteristics in each group were summarized in Table 2. Comparing with controls, stroke patients were less educated with a higher proportion of hypertension, hyperlipidemia, diabetes mellitus, and smoking as well as higher systolic blood pressure at cognitive assessment.

**Table 2:** **Comparison of clinical characteristics**

	**Controls**	**Stroke patients**	***P*** **value**
Age, years	60.4 ± 7.4	62.8 ± 7.8	0.118
Male, no. (%)	24 (48%)	29 (58%)	0.316
Education			0.038
0-6 years	7 (14%)	11 (22%)	-
7-12 years	28 (56%)	33 (66%)	-
>12 years	15 (30%)	6 (12%)	-
Right-handedness, no. (%)	48 (96%)	48 (96%)	1.000
Myocardial infarction, no. (%)	2 (4%)	2 (4%)	1.000
Atrial fibrillation, no. (%)	1 (2%)	1 (2%)	1.000
Hypertension, no. (%)	12 (24%)	39 (78%)	<0.001
Hyperlipidemia, no. (%)	5 (10%)	13 (26%)	0.037
Diabetes mellitus, no. (%)	5 (10%)	18 (36%)	0.002
Smoking, no. (%)	12 (24%)	24 (48%)	0.012
Alcohol abuse, no. (%)	20 (40%)	9 (18%)	0.098
Body mass index, kg/m^2^	24.2 ± 2.5	24.8 ± 3.1	0.270
Systolic blood pressure, mmHg	125.5 ± 18.5	133.6 ± 12.3	0.013
Diastolic blood pressure, mmHg	79.2 ± 10.3	80.9 ± 9.9	0.403
Recurrent stroke, no. (%)	-	16 (32%)	-
NIHSS score	-	2 (0–4)	-

*Abbreviations*: *NIHSS* National Institutes of Health Stroke Scale.

Group comparisons of neuropsychological assessment were shown in Table 3. Stroke patients had significantly lower scores than controls on all individual tests and showed more neuropsychiatric/depressive symptoms, with effect sizes ranging from 0.41 to 1.60. Summary scores of the three neuropsychological protocols in stroke patients were significantly lower than controls (F statistics: 31.1, 31.4 and 24.9 for the 60-min, 30-min and 5-min protocol, respectively, *P* <0.001).

**Table 3:** **Comparison of neuropsychological assessment**

**Cognitive tests**	**Controls**	**Stroke patients**	***P*** **value** ^**b**^	**Cohen’s** ***d***
Executive/Activation				
ANT	17.2 ± 4.0	11.9 ± 4.1	<0.001	1.31
WAIS-III Digit symbol-coding	22.4 ± 7.9	3.4 ± 6.6	<0.001	1.24
TMT A time (sec)	45.7 ± 15.5	90.7 ± 59.1	0.001	1.04
TMT B time (sec)	108.2 ± 42.3	176.4 ± 97.4	0.009	0.92
Domain z score		−1.01		
Language				
Modified BNT	11.4 ± 2.2	8.8 ± 2.7	0.003	1.03
Domain z score		−0.92		
Visuospatial				
RCFT copy	34.6 ± 1.8	30.2 ± 6.9	0.002	0.88
Domain z score		−0.80		
Memory				
HVLT-R delayed recall	7.6 ± 2.6	4.5 ± 2.7	<0.001	1.16
RCFT delayed recall	18.6 ± 6.3	11.9 ± 8.8	<0.001	0.89
Domain z score		−0.93		
Neuropsychiatric/depressive symptoms				
NPI-Q^a^	2 (0–11)	4 (0–29.5)	0.008	0.41
GDS^a^	2 (1–3)	3 (1–7.25)	0.024	0.63
Supplemental tests				
MMSE	28.6 ± 1.1	26.3 ± 3.2	<0.001	0.94
MoCA-BJ	23.8 ± 2.9	17.9 ± 4.4	<0.001	1.59
Protocol summary scores				
60-min	0.46 ± 0.45	−0.49 ± 0.72	<0.001	1.57
30-min	0.53 ± 0.67	−0.53 ± 0.65	<0.001	1.60
5-min	0.41 ± 0.47	−0.42 ± 0.71	<0.001	1.38

^a^Data shown in median (Interquartile range); ^b^Model controlled for years of education.

*Abbreviations*: *ANT* Animal naming test, *TMT* Trail making test, *BNT* Boston naming test, *RCFT* Rey-Osterrieth Complex Figure Test, *HVLT-R* Revised Hopkins verbal learning test, *NPI-Q* Neuropsychiatric Inventory questionnaire, *GDS* Geriatric Depression Scale, *MMSE* Mini-Mental State Examination, *MoCA-BJ* Montreal Cognitive Assessment-Beijing version.

**Figure 1:**
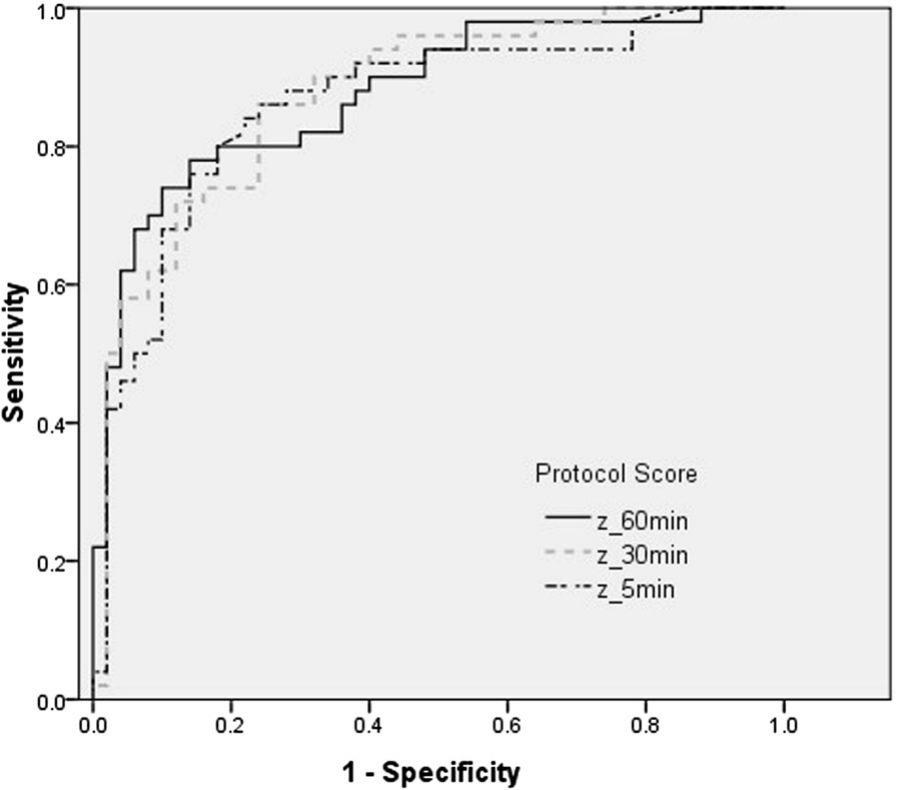
**ROC curves of the three adapted Chinese versions of the NINDS-CSN VCI protocols in discriminating stroke patients from controls.**

External validity evaluated by AUC for the 60-min protocol was 0.88 (95% confidence interval [CI], 0.81-0.95), for the 30-min protocol was 0.88 (95% CI, 0.81-0.94), and for the 5-min protocol was 0.86 (95% CI, 0.79-0.94). AUCs of MoCA-BJ and MMSE were 0.88 (0.81-0.95) and 0.75 (0.65-0.85), respectively. Sensitivity and specificity for various summary z scores of the three NINDS-CSN neuropsychological protocols were shown in Table 4. High sensitivity with relatively good specificity, the highest sum of sensitivity and specificity, and high specificity with relatively good sensitivity were given. A cut-off value of z = −0.014 was optimal for the 60-min protocol, with sensitivity of 74% and specificity of 90%. ROC curves for the three protocol summary scores were presented in Figure 1.

**Table 4:** **Sensitivity and specificity (%) for various z scores of the NINDS-CSN neuropsychological battery**

**NINDS-CSN protocol**	**Summary z score**	**Sensitivity**	**Specificity**
60-min	−0.222	62.0	96.0
	−0.014	74.0	90.0
	0.412	90.0	60.0
30-min	−0.372	58.0	96.0
	0.103	86.0	76.0
	0.393	94.0	60.0
5-min	0.122	76.0	86.0
	0.227	86.0	76.0
	0.379	90.0	66.0

Based on the MMSE and MoCA cut-off points, 12% of patients were considered to be cognitively impaired on the MMSE, whereas 90% of patients were impaired on the MoCA-BJ. MMSE identified 19.4% of the patients who were cognitively impaired by the 60-min protocol, and MoCA-BJ identified 96.8%. Kappa statistic values were 0.030 (*P* = 0.384) between MMSE and MoCA-BJ, 0.154 (*P* = 0.041) between the 60-min protocol and MMSE, and 0.208 (*P* = 0.041) between the 60-min protocol and MoCA-BJ.

Cronbach’s alpha of the cognitive tests was 0.87 for all subjects. Based on a repeated rating after a mean duration of 63.6 days (SD = 27.9) in 12 subjects (2 stroke cases and 10 controls), intra-rater reliability as measured by ICC (95% CI) was 0.90 (0.66-0.97) for the 60-min protocol, 0.83 (0.41-0.95) for the 30-min protocol, and 0.75 (0.14-0.93) for the 5-min protocol.

## Discussion

The adapted Chinese versions of three NINDS-CSN neuropsychological protocols were valid in discriminating stroke patients from cognitively normal controls, with good reliability of reproduction and internal consistency.

The lack of satisfactory criteria for VCI diagnosis and its preventable nature urged the establishment of NINDS-CSN neuropsychological battery [7]. International effort has been made by validation studies from France [9], Hong Kong [8], and Singapore [10]. Our study was consistent with previous studies, also showing good validity and reliability of the protocols for diagnosing VCI. In comparison with our previous Hong Kong study [8], both studies were derived from Chinese patients, however, our previous Hong Kong study validated the protocols in Cantonese, whereas our Chinese versions were adapted in Mandarin Chinese, and modifications were made to TMT, BNT, and the HVLT-R according to the culture in mainland China. Moreover, we provided reliability data in our study which gave more information for assessing VCI in stroke patients.

As expected, stroke patients had a higher proportion of vascular risk factors, and showed poorer cognitive performance and more neuropsychiatric/depressive symptoms than controls. A similar conclusion was drawn by researchers in the Singapore [10] and our previous Hong Kong [8] validation studies. The French study [9] (ClinicalTrials.gov ID: NCT01339195) was supposed to end in August 2013, yet no results have been published so far.

In China, more than 2 million people had vascular dementia [11] with a prevalence of 1.5% in people more than 65 years of age [30]. Stroke doubles the risk of incident dementia [31], thus, it is important to assess post-stroke cognitive function. For patients with mild stroke, the Chinese versions can differentiate them from controls very well, with AUCs of 0.88, 0.88 and 0.86 for the 60-min, 30-min and 5-min protocol, respectively. Two previous studies also demonstrated adequate discriminatory power (corresponding AUCs were 0.90, 0.89 and 0.79 in the Singapore study [10]; 0.79, 0.79 and 0.76 in our previous Hong Kong study [8]). Nonetheless, clinicians should pay attention that these protocols were not applicable to stroke patients with severe impairment of vision, language or consciousness [3]. Moreover, those who had little or no experience in using a pen or in drawing (e.g. illiterate elderly persons) were less motivated for written tasks like TMT and RCFT. Further improved versions were required for the illiterate elders.

Patients with impaired cognition on at least one domain can be identified by MMSE and MoCA at a proportion of 19.4% and 96.8%, respectively. However, they both reached a borderline agreement with the 60-min protocol (*P* = 0.041), and MMSE showed a ceiling effect with high scores in stroke patients (mean score, 26.3 ± 3.2), which was also observed in Tombaugh TN, et al. study [32]. Therefore, MoCA could be a more sensitive test than MMSE for VCI screening in post-stroke patients [33].

There were several limitations of our study. First, our cases were patients who suffered relatively mild stroke. Therefore, our results may not be representative of VCI patients with more severe strokes or cognitive disorders related to other vascular brain diseases. However, this on the other hand highlighted the sensitivity of these protocols, for that cognitive impairment can be detected even in patients with mild stroke. Second, the patient group had a higher proportion of hypertension, hyperlipidemia, diabetes mellitus, and smoking, and these factors would impact cognitive performance of the NINDS-CSN battery. However, they had not been matched when choosing the control group, hence, the battery should be applied with caution that in stroke patients with a greater burden of vascular risk factors, a lower reliability and a higher discrimilinatory ability was likely to happen, in consideration of the contributions of cognitive dysfunction from related vascular risk factors. Third, controls were selected based on normal MMSE without excluding substantial small vessel diseases that are common in VCI. Therefore we not completely excluded subjects with mild cognitive impairment in this group. Fourth, the sample size of 50 was determined empirically, with reference to our previous validation study in Hong Kong [8]. However, according to Schmidt et al., if true validity is 0.50, criterion reliability is 0.80, the selection ratio is 1.00, and a two-tailed test is used, a sample size of 49 is required for power 90% [34], thus our sample size would be adequate. Fifth, the intra-rater reliability analysis was based on a sample of only 12 subjects, which would possibly introduce biases towards better consistency. Sixth, controls had higher levels of education than stroke patients, yet this was adjusted in the statistical analyses.

## Conclusions

Our study suggested that the adapted Chinese versions of NINDS-CSN neuropsychological protocols were valid and reliable for assessing VCI in mainland Chinese stroke patients. Our study would contribute to the international effort for the development of VCI common standards, and help early identification and diagnosis of VCI in China.
